# Trauma Revealing a Tumour: A Case Report

**DOI:** 10.7759/cureus.88151

**Published:** 2025-07-17

**Authors:** Allan Zhou, Claire Liegeois, Suraya Zainal Abidin, Eunizar Omar

**Affiliations:** 1 Emergency Medicine, Sengkang General Hospital, Singapore, SGP; 2 Orthopaedics, Singapore General Hospital, Singapore, SGP

**Keywords:** bone neoplasms, case report, diagnostic imaging, giant-cell tumour of bone, pathological fractures

## Abstract

A 25-year-old male patient presented to the emergency department with acute left knee pain and swelling after a fall. He had been experiencing left knee pain for several months. Examination revealed a swollen left knee held in a flexed position with limitation in range of motion. Plain radiographs performed showed a lytic lesion over the left lateral femoral condyle, and the patient was admitted for computed tomography (CT) and magnetic resonance imaging (MRI) scans. An open biopsy was performed, and immunohistochemical staining of the tumour cells was positive for H3G34W, confirming the diagnosis of giant-cell tumour of bone (GCTB). The patient subsequently underwent left distal femur GCTB curettage, bone grafting, and internal fixation. This case highlights how incidental trauma can reveal a previously undiagnosed bone tumour, underscoring the need for clinicians to maintain a high index of suspicion when evaluating patients.

## Introduction

Giant-cell tumours of bone (GCTBs) are typically benign but locally aggressive neoplasms, accounting for approximately 5-7% of primary bone tumours [1]. GCTBs are unpredictable in behaviour, with a spectrum of clinical presentations ranging from contained intraosseous lesions to cases involving extensive bone destruction, recurrence after surgical treatment, and, in rare instances, distant metastases. Pulmonary metastasis has been reported in up to 9% of cases, while malignant transformation, often associated with a poor prognosis, may occur in approximately 4% [2].

GCTBs typically affect adults between the ages of 20 and 40 and are slightly more common in females, with a reported female-to-male ratio of 1.2:1. Although population-level data are limited in many regions, the global incidence is estimated at approximately 1.2 to 1.7 cases per million individuals per year [3].

GCTBs frequently arise at the epiphyseal-metaphyseal regions of long bones, with the knee joint, the distal femur (26%) and the proximal tibia (20%) being the most commonly affected sites. Less frequently, it affects the distal radius, sacrum, spine, pelvis, and short tubular bones of the hands and feet [3,4].

Patients with GCTBs can present with chronic joint pain, visible swelling, and impaired mobility of the affected joint, but some remain asymptomatic or minimally symptomatic until triggered by trauma [5]. We present a case of how incidental trauma brought a patient to the emergency department, ultimately uncovering a history of chronic pain affecting his daily activities.

## Case presentation

A 25-year-old male with no past medical history presented to the emergency department with acute left knee pain and swelling following a fall on a wet floor. He reported hearing a "pop" and was unable to bear weight afterward. The patient had been experiencing left knee pain for several months, which had been worsening for the past few weeks, resulting in him having to stop running. The patient did not report constitutional symptoms of loss of weight and loss of appetite, and he did not have a family history of malignancy.

The clinical examination revealed a grossly swollen left knee that was tender to the touch. There was no overlying skin injury. His left knee was held in a 45° flexed position and unable to range beyond that. The neurovascular status of the left lower limb was intact.

**Figure 1:**
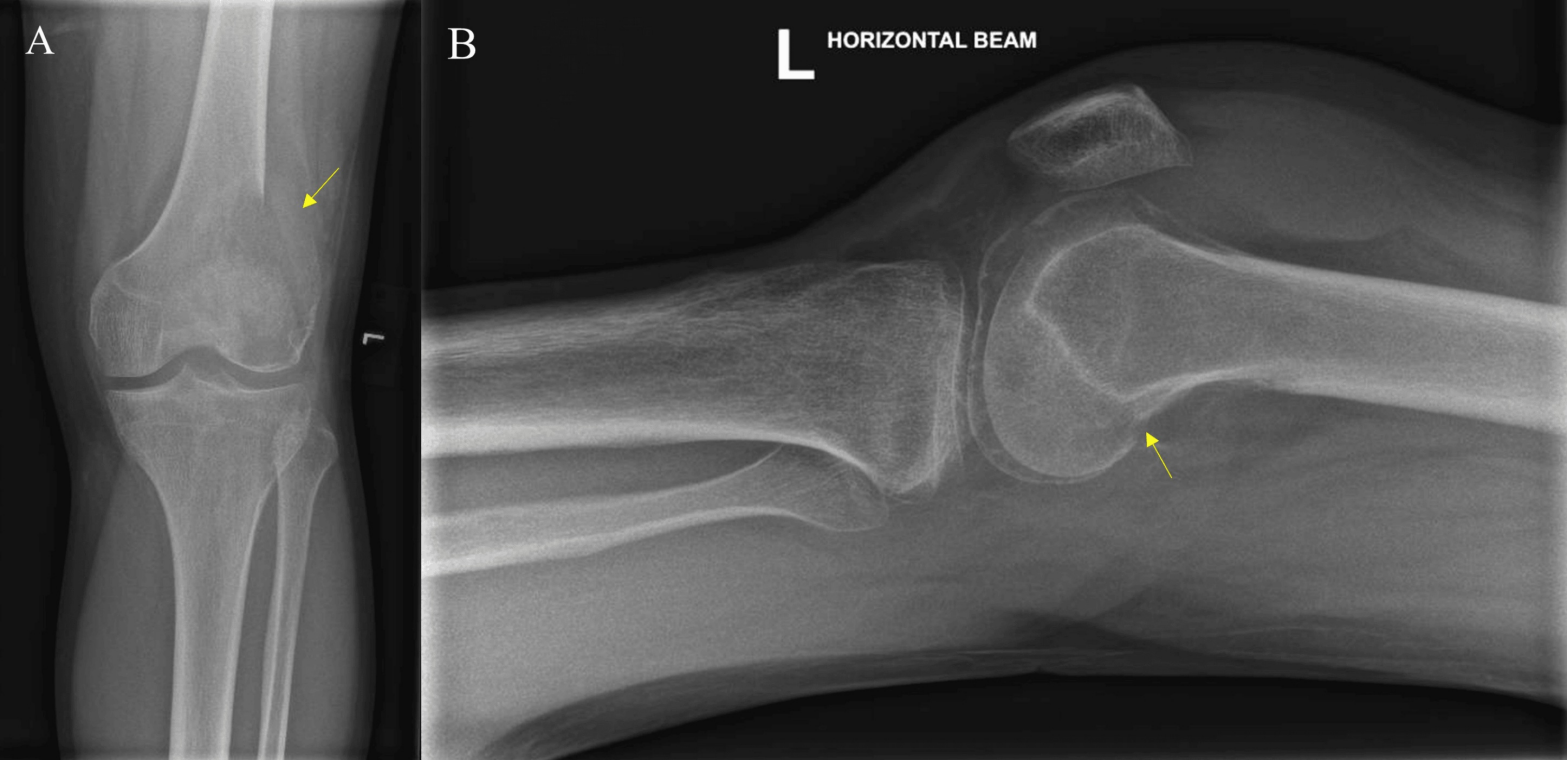
Plain radiographs of the left knee (A) Anteroposterior view identifying a lytic lesion and cortical irregularity over the lateral femoral condyle; (B) lateral view showing a lytic lesion in the distal femur and cortical irregularity over the posterior aspect

Blood investigations performed showed an elevated white cell count of 14.05 × 10⁹/L (normal range: 4.00-10.00 × 10⁹/L), while C-reactive protein level was 4.6 mg/L, within normal limits (normal range: < 4.9 mg/L), consistent with reactive inflammation likely secondary to soft tissue inflammation or recent trauma. The renal and liver panels, as well as calcium and phosphate levels, were within normal limits. Plain radiographs of the left knee (Figure 1) were performed, showing a lytic lesion over the lateral distal femur involving the lateral femoral condyle with cortical irregularity over the lateral femoral condyle. The chest radiograph showed no abnormalities and no evidence of pulmonary metastasis. The patient was admitted for further computed tomography (CT) and magnetic resonance imaging (MRI) scans of the left knee to be performed.

**Figure 2:**
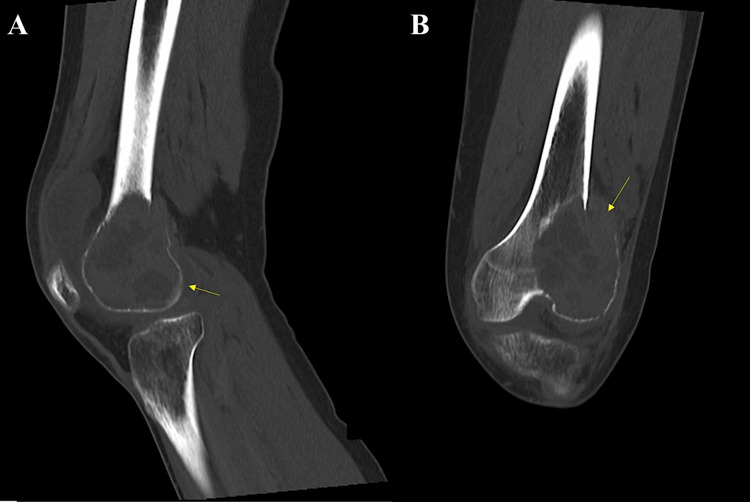
CT images of the left knee (A) Sagittal view showing a transmedullary lesion with cystic changes and pathological fracture over the posterior aspect of the left distal femur; (B) coronal view showing a transmedullary lesion with cystic changes and pathological fracture involving the lateral femoral condyle

CT of the left knee (Figure 2) confirmed a trans-medullary lesion showing areas of cystic change with multiple fluid levels and pathological fracture of the distal left femur laterally and the lateral femoral condyle.

**Figure 3:**
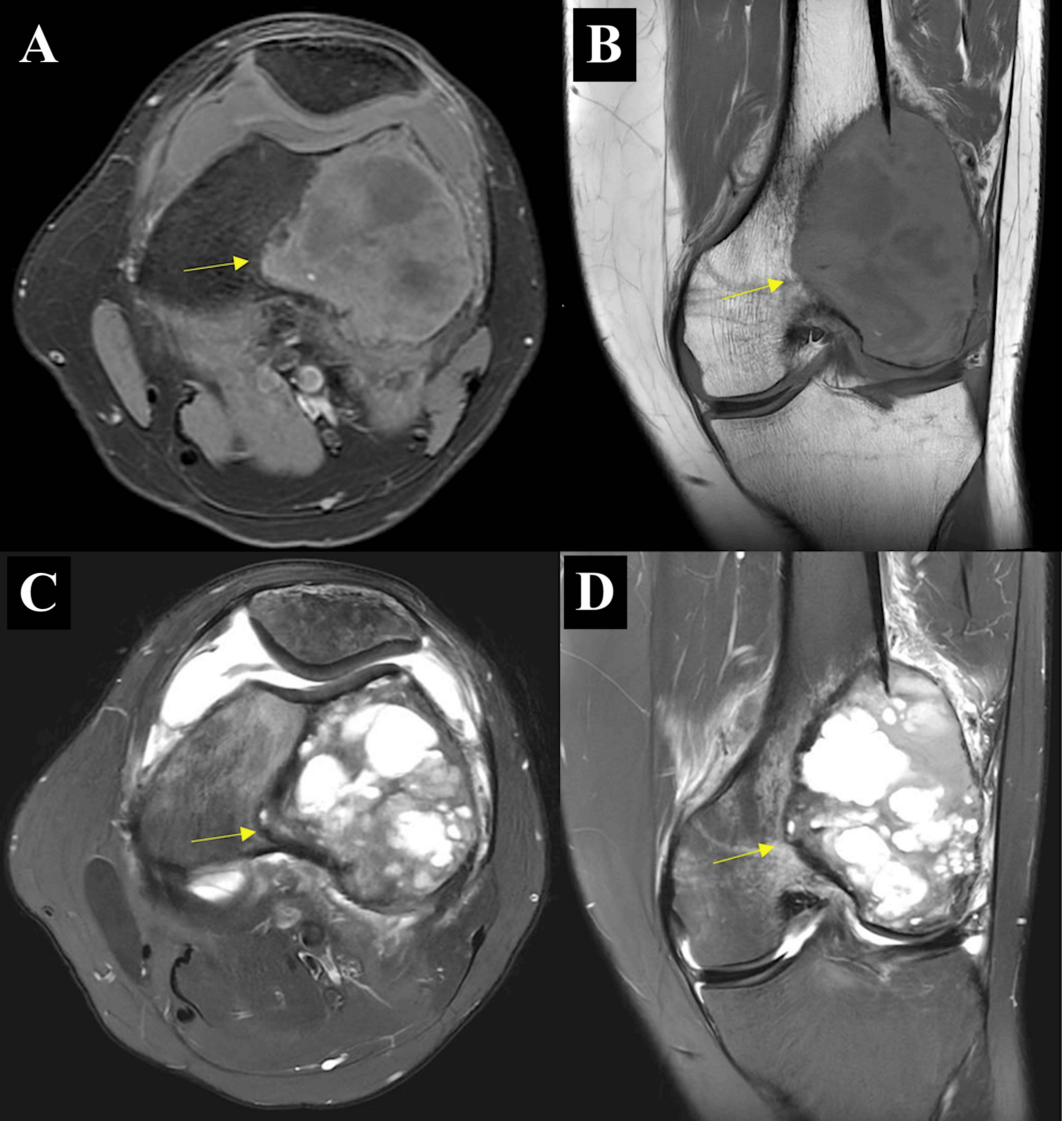
MRI images of the left distal femur T1 and T2 MRI images showing a lesion over the lateral femoral condyle with solid and cystic components, demonstrating low to intermediate T1-weighted signal and high to intermediate T2-weighted signal. (A) T1-weighted axial view; (B) T1-weighted coronal view; (C) T2-weighted axial view; (D) T2-weighted coronal view

MRI of the left knee (Figure 3) further defined the 5.7 × 6.0 × 7.4 cm expansile lesion over the lateral femoral condyle, which had both solid and cystic components. The lesion exhibits low to intermediate T1-weighted signal intensity and high to intermediate T2-weighted signal intensity.

The differentials for an expansile bone lesion in the lateral femoral condyle include giant cell tumour, aneurysmal bone cyst, and other malignant lesions such as osteosarcoma and chondroblastoma.

**Figure 4:**
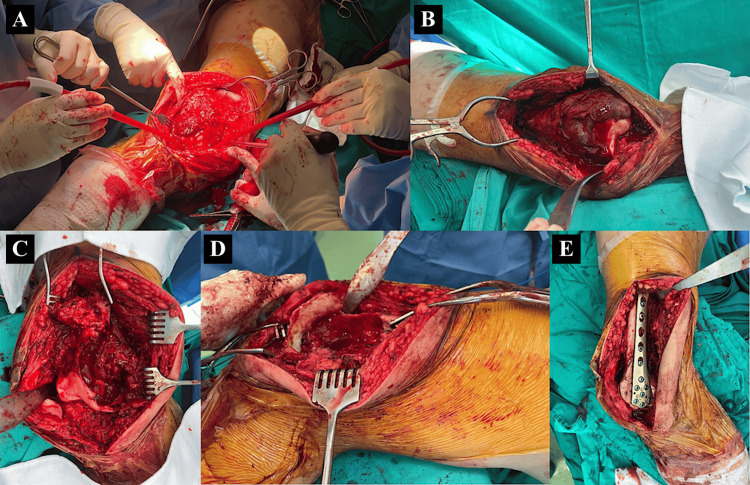
Intraoperative images Intraoperative images demonstrating key steps in the surgical management of a giant cell tumour of the distal femur. (A) Exposure of the distal femur; (B) identification of the tumour within the lateral femoral condyle (circled); (C, D) lesion cavity post-curettage; (E) internal fixation with plate following curettage and bone grafting

Open biopsy was performed, and immunohistochemical staining of the tumour cells was positive for H3G34W, confirming the diagnosis of GCTB. The patient subsequently underwent left distal femur GCTB curettage, bone grafting, and internal fixation (intraoperative images attached as Figure 4).

Postoperatively, the patient was initially on crutches and underwent regular physiotherapy sessions. He was eventually transitioned to ambulating with a walking stick. At review two months after the operation, the patient was ambulating independently and painlessly, with a left knee range of motion of 5-90°. Plain radiographs of the left knee showed stable surgical implants with no radiological evidence of tumour recurrence. The patient was encouraged to continue rehabilitation exercises to further improve his range of motion.

## Discussion

Imaging with plain radiographs, CT, and MRI is indicated in the workup of all patients presenting with suspicious bony lesions or persistent joint pain. Radiographic imaging typically reveals an expansile, lytic lesion with a possible cortical breach. MRI further delineates soft tissue involvement and may demonstrate fluid-fluid levels in the presence of secondary aneurysmal bone cyst components [6].

Besides imaging, histology is also important to distinguish GCTBs from other radiological differentials and for differentiating subtypes. Histologically, GCTBs consist of mononuclear stromal cells and multinucleated osteoclast-like giant cells. Immunohistochemistry for the H3G34W mutation supports the diagnosis and helps distinguish GCTB from histological mimics such as chondroblastoma [2].

GCTBs are classified radiographically into three grades using the Campanacci system. Grade I lesions are well-marginated and confined within an intact cortex, Grade II lesions show thinning and expansion of the cortex without breakthrough, and Grade III lesions demonstrate cortical destruction with soft tissue extension [4].

Surgical treatment is the mainstay of treatment and is indicated for accessible lesions to achieve local tumour control. The type of surgery depends on the tumour subtype. Grades I and II GCTBs can be managed with intralesional curettage and en bloc resection, whereas Grade III GCTBs would require wide resection. The aim is to preserve as much bone as possible for the best functional outcome, but local recurrence rates can reach up to 35-50% [5].

Denosumab, a monoclonal antibody targeting RANKL, is now used in select cases of unresectable, recurrent, or surgically morbid GCTBs. While effective in downstaging tumours, it remains investigational in terms of long-term control and recurrence risk [7]. In this case, denosumab was not used as the tumour was amenable to surgical resection.

The prognoses of GCTBs are generally favourable, particularly for benign lesions treated early. However, long-term outcomes depend on tumour grade and the presence of metastasis. Intralesional curettage offers joint preservation but has a recurrence risk of 35-40%, whereas en bloc resection reduces recurrence to 0-12% at the expense of greater surgical morbidity and poorer functional outcomes [2].

## Conclusions

Emergency physicians need to maintain a high index of suspicion when managing cases of trauma. This case highlights how incidental trauma can reveal a previously undiagnosed bone tumour, emphasising the importance for clinicians to take a thorough history to pick up on chronic symptoms preceding the traumatic event that may point to the presence of an underlying, more sinister pathology.

## References

[1] Goldenberg RR, Campbell CJ, Bonfiglio M (1970). Giant-cell tumor of bone: an analysis of two hundred and eighteen cases. J Bone Joint Surg Am.

[2] Abbasi AN, Qamar J, Habib A, Ali SME, Ahmed S, Khan MW (2025). Unraveling the mystery: a comprehensive review of multidisciplinary strategies for managing giant cell tumor of the bone. J Orthop Rep.

[3] Parmeggiani A, Miceli M, Errani C, Facchini G (2021). State of the art and new concepts in giant cell tumor of bone: imaging features and tumor characteristics. Cancers (Basel).

[4] Campanacci M, Baldini N, Boriani S, Sudanese A (1987). Giant-cell tumor of bone. J Bone Joint Surg Am.

[5] Blackley HR, Wunder JS, Davis AM, White LM, Kandel R, Bell RS (1999). Treatment of giant-cell tumors of long bones with curettage and bone-grafting. J Bone Joint Surg Am.

[6] Murphey MD, Nomikos GC, Flemming DJ, Gannon FH, Temple HT, Kransdorf MJ (2001). Imaging of giant cell tumor and giant cell reparative granuloma of bone: radiologic-pathologic correlation. Radiographics.

[7] Chawla S, Henshaw R, Seeger L (2013). Safety and efficacy of denosumab for adults and skeletally mature adolescents with giant cell tumour of bone: interim analysis of an open-label phase 2 study. Lancet Oncol.

